# Dr. R. N. Sharma

**DOI:** 10.4103/0970-0358.59270

**Published:** 2009

**Authors:** Arun Kumar Singh

**Affiliations:** Department of Plastic & Reconstructive Surgery, King George's Medical College, Lucknow, India. E-mail: singhkarun@hotmail.com

**Figure:**
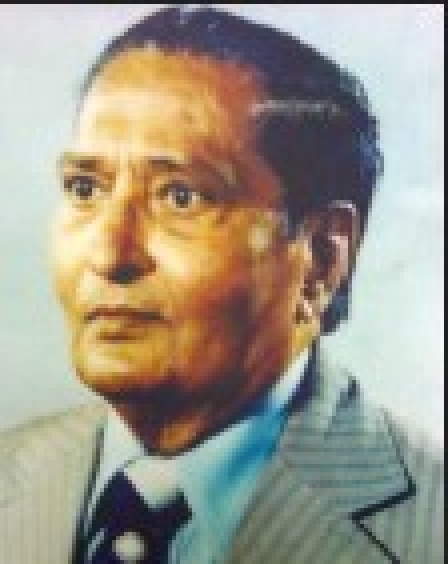
Dr. R. N. Sharma

Dr. Ravindra Nath Sharma, MS. FRCS was head of the P.G. Department of Plastic Surgery of King George's, Lucknow and was one of the pioneers for the development of Plastic Surgery in India.

He worked with Mr. Pomfret Kilner in 1949 at Stoke Mandeville Hospital Aylesbery, UK while on a Common wealth Fellowship. On his return from UK in 1953 with a dream to develop a Plastic Surgery unit in Lucknow, he whole heartedly worked towards this objective, giving up his rights to do General Surgery in his parent department. It was due to his untiring efforts, punctuated by surgical brilliance that he succeeded in convincing one and all of the importance of an independent department, with its own outpatient department, wards operating theatres and emergencies. Since 1966, regular MCh programme (earlier MS Plastic Surgery) was started in this Unit. A pioneer of structured training in plastic surgery, many of his residents today head such programmes as Professorss & Head around the globe, carrying his legacy forward and propagating the science and art of Plastic Surgery. Dr. Sharma retired in 1982, after a long and dedicated service to the specialty.

He was a dynamic visionary. He realized the importance of team work and transformed the department from dependency to self-sufficiency. The department he headed, prided itself with possessing, right from its inception, its own team of orthodontist, anaesthetiest, prosthetist, speech therapist and physiotherapist. The department possessed a prosthetic laboratory, a speech centre, and a splint fabrication laboratory. In 1976, when microscopic reconstructive surgery was not yet popular, he procured two operating microscopes. He fondly talked about the day when Plastic Surgeons and Neurosurgeons would work together to treat severe orbito-facial birth defects and tumours infiltrating the skull base! He was thus surely far ahead of his time, with a 20-20 vision into the future! Besides being a brilliant academician, he was deeply involved in cultural activities of the college and was the President of the Dramatics Society.

He was a meticulus surgeon and a master craftsman. He would not hesitate to reopen the lip (even as the anesthesiologist was preparing to extubate the patient), if he thought it was not a perfect repair. He was a hard task master, but extremely gentle at heart and always ready to help students, colleagues and employees. At the end of the day, everyone realized that his stern veneer concealed his hallmark humane and pious nature, forever ready to help people, while continuously striving to acquire and disseminate knowledge towards attaining the goal of perfection in the field of plastic surgery.

Despite today's state of the art techniques, assisted with technology, some of his surgical results are difficult to reproduce. The return of continence in ectopia vesicae, full mandible reconstruction with non-vascularised rib grafts, good speech in cleft patients, and good aesthetic reconstructions, are an ode to his surgical skills and set him apart as a colossus who strode tall during his times.

He was a founder executive member of the Association of Plastic Surgeons of India of which he became the president in 1967. He participated in several international meetings and was invited as a visiting faculty to number of units abroad. Many eminent plastic surgeons like Eric Peet, Schilli, Mustarde, Bruce Bailey, Hunter, Semple, to name a few, visited him and were amazed by his industrious zeal towards establishing Plastic Surgery in India.

Reminiscing after his retirement, Prof RN Sharma wrote in his memoir, “Readers will agree that it is not possible to attain everything that one would like to, in one's life time. However, I have tried my best to discharge my responsibilities and was able to train twenty one post graduates who are now working in India and abroad.”

